# Compact Inner-Wall Grating Slot Microring Resonator for Label-Free Sensing

**DOI:** 10.3390/s19225038

**Published:** 2019-11-19

**Authors:** Hongjun Gu, He Gong, Chunxue Wang, Xiaoqiang Sun, Xibin Wang, Yunji Yi, Changming Chen, Fei Wang, Daming Zhang

**Affiliations:** 1 State Key Laboratory of Integrated Optoelectronics, College of Electronic Science and Engineering, Jilin University, 2699 Qianjin Street, Changchun 130012, Chinawangcx2019@139.com (C.W.); sunxq@jlu.edu.cn (X.S.); xibinwang@jlu.edu.cn (X.W.); yiyj@jlu.edu.cn (Y.Y.); Chencm@jlu.edu.cn (C.C.); wang_fei@jlu.edu.cn (F.W.); 2 College of Information Technology, Jilin Agricultural University, 2888 Xincheng Street, Changchun 130118, China

**Keywords:** microring resonator, inner-wall grating, slot waveguide, label-free, bulk sensing

## Abstract

In this paper, we present and analyze a compact inner-wall grating slot microring resonator (IG-SMRR) with the footprint of less than 13 μm × 13 μm on the silicon-on-insulator (SOI) platform for label-free sensing, which comprises a slot microring resonator (SMRR) and inner-wall grating (IG). Its detection range is significantly enhanced without the limitation of the free spectral region (FSR) owing to the combination of SMRR and IG. The IG-SMRR has an ultra-large quasi-FSR of 84.5 nm as the detection range, and enlarged factor is up to over 3 compared with the conventional SMRR. The concentration sensitivities of sodium chloride solutions and D-glucose solutions are 996.91 pm/% and 968.05 pm/%, respectively, and the corresponding refractive index (RI) sensitivities are 559.5 nm/RIU (refractive index unit) and 558.3 nm/RIU, respectively. The investigation on the combination of SMRR and IG is a valuable exploration of label-free sensing application for ultra-large detection range and ultra-high sensitivity in future.

## 1. Introduction

Label-free optical sensors have been investigated extensively in many applications, such as medical diagnostics, drug detection, food security, pesticide residue detection, environmental monitoring, homeland defense, and so on. In the optical sensing applications, two detection strategies, label-based detection and label-free detection are implemented [1]. By comparison, the former suffers from the complex labeling procedures and relatively long assay time, and the latter can be chosen as an alternative for relatively easy and cheap sensing scenarios [2].

In recent decades, a silicon-on-insulator (SOI) platform has been recognized as a favorable candidate due to its compatibility with well-established complementary metal oxide semiconductor (CMOS) manufacturing technology. SOI waveguide can offer high refractive index (RI) contrast that permits strong light mode field confinement and compact bends (down to 1.5 μm bending radius approaching the theory limit) [3]. The optical sensing devices based on the SOI platform have been widely studied, such as Mach–Zehnder interferometer sensors [4], Fabry–Perot resonance sensors [5], surface plasmon sensors [6,7], microring/microdisk resonator sensors [8,9,10,11], and grating sensors [12]. The microring resonator (MRR) with high qualify factor (Q-factor) enables lights to circle the rings scores of times before being lost, which provides an equivalently long light-matter interaction distance. Therefore, the attractive sensitivity of the optical MRR sensor can be achieved. In addition, the MRR sensor with smaller footprints needs less amount of analyte and is easily integrated in the sensing arrays.

For MRR sensor, two typical interrogation approaches, intensity interrogation and wavelength interrogation [13], have been utilized. The detection range of the former is too small, which is suitable for the relatively lower RI variation of analyte. The latter as the popular detection method can satisfy the actual production demand. The low sensitivity of MRR sensor based on traditional strip waveguide is around 70 nm/RIU [14], and the reason is that the lights trapped in the SOI waveguide cores cannot interact fully with the matter. The SMRR with much light in the slot can enhance the light–analyte interaction. Hence, the SMRR sensor has higher sensitivity. However, the detection range of conventional MRR sensor based on the wavelength shift is severely constrained by the small free spectral region (FSR). In order to enlarge the detection range, some schemes, such as serially coupled double MRRs [15], the MRR with bent contra-directional couplers [16], Mach–Zehnder interferential couple MRR [17], grating-coupled silicon MRR [18], and angular grating MRR [19] are investigated to expand the FSR. These schemes can enlarge the FSR, but the sensitivities of the above schemes are relativity lower than the sensitivity of SMRR.

In this paper, we present a compact optical label-free sensor based on IG-SMRR to acquire the ultra-large detection range and ensure the high sensitivity. The sensor adopts all-pass filter SMRR, in the inner-wall of which is integrated by a grating on an SOI platform. Lumerical MODE Solutions as a popular simulation software for fiber optics and integrated photonics is utilized to simulate the related parameters and sensing performance of the device. The relations between the side mode suppression ratio (SMSR), the extinction ratio (ER), the Q-factor, and the structural parameters are investigated. Taking the sodium chloride solutions and D-glucose solution as the top cladding layer, the sensing characteristics of the optical label-free sensor are demonstrated.

## 2. Structure Design and Operation Principle

### 2.1. Structure Design

**Figure 1:**
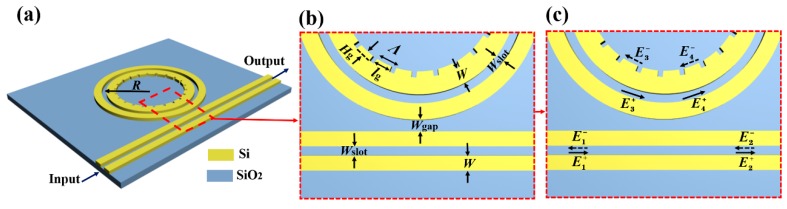
(**a**) Three-dimensional (3D) schematic of the proposed device. (**b**) Geometric parameters of the IG-SMRR in the coupling region. (**c**) Propagation of the optical fields. IG-SMRR: slot microring resonator and inner-wall grating.

The three-dimensional (3D) schematic of the proposed sensing device is shown Figure 1a. The SOI wafer was adopted as the waveguide material, with 220 nm Si on a 2 μm SiO_2_ substrate. Homogeneous sensing is implemented in this paper, so the sensor device is immersed in aqueous solutions. Naturally, pure water was chosen as the top cladding in the process of determining the geometric parameters of waveguides. This homogeneous sensing case can be easily extended to surface sensing applications by covering thin adsorbed analyte for the top cladding in the calculation. The bent radius (*R*) of the SMRR is designated as the distance between the center of the rings and the middle of the slot, and set to 5.86 μm. The gap width between the bus and the ring waveguide is denoted as *W*_gap_. Other geometric parameters are depicted in Figure 1a,b. The ring and bus waveguide have the same slot width (*W*_slot_). The strip waveguide width *W* and the slot width *W*_slot_ are set to 210 nm and 100 nm, respectively, which enables an extremely strong restriction of the electric field with the mode confinement factor of over 30% [20]. The etched IG has the azimuthal period (*Λ*) of about 1171 nm, azimuthal width (*l*_g_), and the duty cycle (*F*) (ratio of silicon block to the period). The structure of the gratings is achieved by etching a quasi-rectangular region (*l*_g_ × *H*_g_) from the inner-wall of the ring waveguide. Here, *H*_g_ is corrugation depth of the grating. Propagation of the optical fields is shown in Figure 1c. Here, $E_{1}^{+}$, $E_{2}^{+}$, $E_{3}^{+}$, $E_{4}^{+}$ represent incident optical field amplitudes, and $E_{1}^{-}$, $E_{2}^{-}$, $E_{3}^{-}$, $E_{4}^{-}$ represent reflected optical field amplitudes in the corresponding positions.

### 2.2. Operation Principle

The resonance equation of SMRR can be expressed:

(1) $$Ln_{\mathrm{eff}}=m\lambda_{\mathrm{res}},m=1,2,3,......$$

where, *L = 2πR* is the perimeter of the ring, *n*_eff_ is effective RI, m (positive integer) is the azimuthal resonant order, and *λ*_res_ is the resonant wavelength.

The input and output ports of the IG-SMRR device are symmetric, so the optical path is reversible. The transmission matrix of incident optical fields between the bus waveguide and ring waveguide is expressed as

(2) $$\left(\begin{array}{c} E_{2}^{+}\\ E_{4}^{+} \end{array}\right)=\left(\begin{array}{cc} \tau & j\kappa \\ j\kappa & \tau \end{array}\right)\left(\begin{array}{c} E_{1}^{+}\\ E_{3}^{+} \end{array}\right),$$

where, κ and τ are amplitude coupling coefficient and amplitude transmission coefficient, respectively. When coupling loss can be ignored, the relation between them is described as

(3) $$\kappa^{2}+\tau^{2}=1.$$

Similarly, the transmission matrix of reflected optical fields is denoted as

(4) $$\left(\begin{array}{c} E_{1}^{-}\\ E_{3}^{-} \end{array}\right)=\left(\begin{array}{cc} \tau & j\kappa \\ j\kappa & \tau \end{array}\right)\left(\begin{array}{c} E_{2}^{-}\\ E_{4}^{-} \end{array}\right).$$

The transmission matrix of inner-wall grating [21] is written as

(5) $$\left(\begin{array}{c} E_{3}^{+}\\ E_{4}^{-} \end{array}\right)=e^{-\alpha L}\cdot e^{j\beta L}\cdot S\left(\begin{array}{c} E_{3}^{-}\\ E_{4}^{+} \end{array}\right)=e^{-\alpha L}\cdot e^{j\beta L}\cdot \left(\begin{array}{cc} -jre^{-j\varphi } & t\\ t & jre^{-j\varphi } \end{array}\right)\cdot e^{j\phi }\left(\begin{array}{c} E_{3}^{-}\\ E_{4}^{+} \end{array}\right),$$

where, *S* is the scattering matrix of inner-wall grating, *α* and *β* are transmission loss coefficient and propagation constant, respectively. When transmission loss is negligible, then *α* = 0. In the S matrix, *r* and *t* are reflected coefficient and transmission coefficient of inner-wall grating, respectively. And *φ* and *ϕ* are the phases of *r* and *t,* respectively. The parameters *r* and *t* satisfy

(6) $$k^{2}+t^{2}=1.$$

Combining (2), (3), (4), (5), (6), and $E_{2}^{-}$ = 0 (no input signal for output port), the transfer function of IG-SMRR is derived as

(7) $$A={\left|\frac{E_{2}^{+}}{E_{1}^{+}}\right|}^{2}=\frac{{\left[t(1+\tau^{2})-2\tau \cos\left(\beta L+\phi \right)\right]}^{2}}{\left(1-t^{2}\right)\left[{\left(1+\tau^{2}\right)}^{2}-4\tau^{2}\right]+{\left[t(1+\tau^{2})-2\tau \cos\left(\beta L+\phi \right)\right]}^{2}}$$

which is usually expressed as the logarithmic form

(8) $$T(\lambda )=10_{\log10}(A).$$

**Figure 2:**
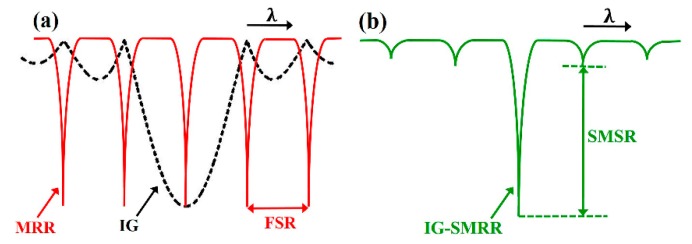
Demonstration of the operating principle. (**a**) The spectral responses of the SMRR (slot microring resonator) and the IG. (**b**) The spectral response of the IG-SMRR.

The operating principle of the designed sensor is demonstrated in Figure 2, in which the spectral responses of SMRR, IG, and IG-SMRR are described. The optical transmission can be regarded as two filtering processes. The light in the bus waveguide is filtered by SMRR at first, and the resonant light is filtered by IG again. SMRR and IG have the same resonant wavelength (near @1550 nm) as the main resonant peak of IG-SMRR, and other resonant lights of SMRR are filtered by IG. The IG-SMRR with the wavelength-selective characteristic is not restricted by the FSR of SMRR. In the second filtering course, some side-modes should be effectively suppressed by optimizing the corrugation depth of IG. Side-mode suppression ratio (SMSR) is utilized to evaluate the suppressed quality.

## 3. Results and Discussion

**Figure 3:**
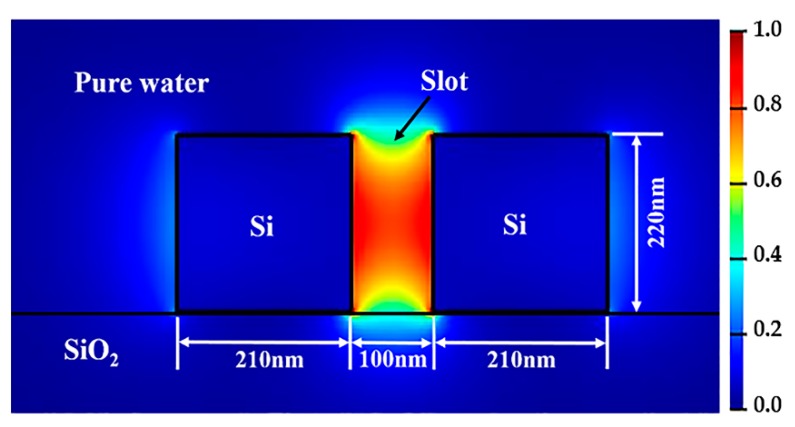
Mode field distribution of the slotted waveguide.

MODE Solutions software of Lumerical Inc. (Vancouver, BC, Canada) [22] was utilized to construct the device model and calculate the spectral responses of the sensor. A tunable laser of TE-like (the fundamental mode, TE_0_) was injected into the bus waveguide. The mode field distribution was calculated by using Finite Difference Eigenmode (FDE) solver, as shown in Figure 3. The varFDTD solver based on variational FDTD propagation method collapses a 3D geometry into a two-dimensional (2D) set of effective indices that can be solved with 2D FDTD (usually regarded as 2.5D), which ensures the high calculation accuracy and saves much memory and simulation time. The spectral responses of the proposed sensor were carried out by the varFDTD solver. In the process of the following parameter optimization, pure water is acted as the top cladding.

The output spectra can be expressed as:

(9) $$T=10lg\frac{P_{\mathrm{out}}}{P_{\mathrm{in}}}$$

where *P*_in_ and *P*_out_ are the power flow integrals at the input and output ports, respectively. Q-factor can be calculated from the expression:

(10) $$Q-\mathrm{factor}=\frac{\lambda_{res}}{3\mathrm{dB}\;\mathrm{bandwidth}}.$$

### 3.1. Optimization of Parameters

High sensitivity of the optical label-free sensor is vital in the practical measuring application. Extinction ration (ER) and Q-factor are two key parameters, which determine the transmission spectrum properties of IG-SMRR and further influence the sensitivity. Intrinsic propagation loss and coupling loss are main loss resources of the IG-SMRR. SOI waveguide can neglect radiation loss bending loss for *R* > 3 μm owing to high index contrast [23]. The scattering power loss is mainly caused by IG (less than 13%) [24]. In this case, the coupling loss and scattering loss dominate the total loss, and a small coupling distance can effectively decrease coupling loss [25]. 

In the following parameter optimizations, the variables duty cycle *F*, corrugation depth *H*_g_, and coupling distance *W*_gap_ are required to be determined. Firstly, the influence of *F* and *H*_g_ on IG-SMRR is investigated at the *W*_gap_ of 180 nm, and suitable values can be selected. To decrease the scattering loss of IG, the principle of choosing *F* and *H*_g_ tends to pursue for high Q-factor. After *F* and *H*_g_ are designated as reasonable values, *W*_gap_ is optimized by considering Q-factor and ER of the device.

**Figure 4:**
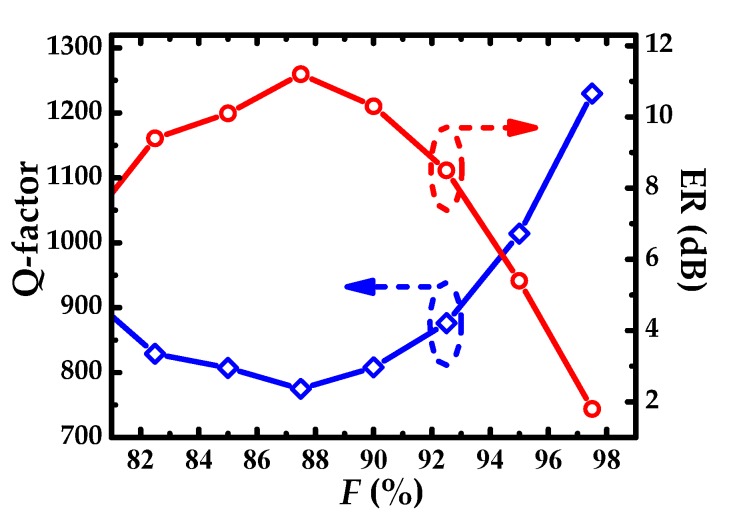
Qualify factor (Q-factor) and extinction ration (ER) as a function of duty cycle *F.*

The dependence of Q-factor and ER on the duty cycle *F* are shown in Figure 4. Variation trend of Q-factor and ER is in the opposite direction. Considering the trade-off between Q-factor and ER, *F* is set to 95%, and the corresponding Q-factor and ER are 1014 and 5.4 dB, respectively. Definitely, ER can be enhanced by balancing the corrugation depth *H*_g_ and adjusting the coupling distance *W*_gap_. When *F* increases to 97.5%, ER less than 2 is difficult to be optimized to a favorite value in the subsequent parameter optimization. 

**Figure 5:**
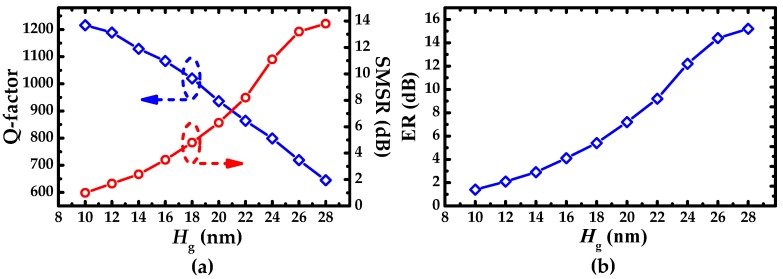
(**a**) Q-factor and side mode suppression ratio (SMSR) and (**b**) ER as a function of etched depth *H*g of IG.

Q-factor, SMSR, and ER as a function of etched depth *H*_g_ of the IG are shown in Figure 5a,b. Q-factor decreases linearly with the increase of *H*_g_, which is due to the increasing of scattering loss caused by IG. But bigger *H*_g_ is beneficial to SMSR and ER. Therefore, considering the trade-off of Q-factor and SMSR, *H*_g_ was chosen as 18 nm. The corresponding Q-factor, ER, and SMSR are 1020, 5.4 dB, and 4.8 dB, respectively. Admittedly, the small size of *H*_g_ brings a high degree of difficulty for the device fabrication. W. Shi et al. fabricated the sidewall grating with the corrugation depth of 15 nm based on SOI platform and achieved the wide operating range for MRR.

**Figure 6:**
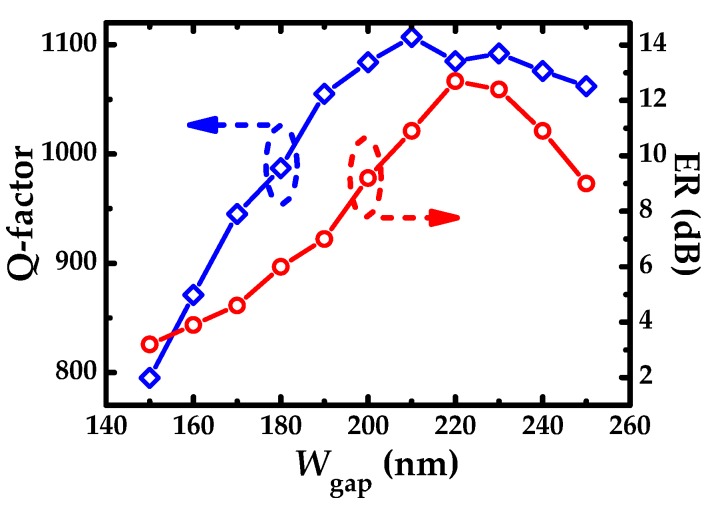
Q-factor and ER as a function of coupling distance *W*_gap._

Figure 6 plots Q-factor and ER as a function of coupling distance *W*_gap_ (*F* = 95%, *H*_g_ = 18 nm). Q-factor increases rapidly, then tends to fluctuate slightly. ER increases first, and then levels off gradually after reaching the maximum, and at last, manifests a downward trend. In the whole process, the operating states of SMRR transit from over coupling to critical coupling with the increase of ER, and from critical coupling to under coupling with the decline of ER. *W*_gap_ = 220 nm is favorable after trading off ER, Q-factor, and coupling loss, the corresponding ER, SMSR, and Q-factor are 12.7 dB, 11.4 dB and 1085, respectively.

**Figure 7:**
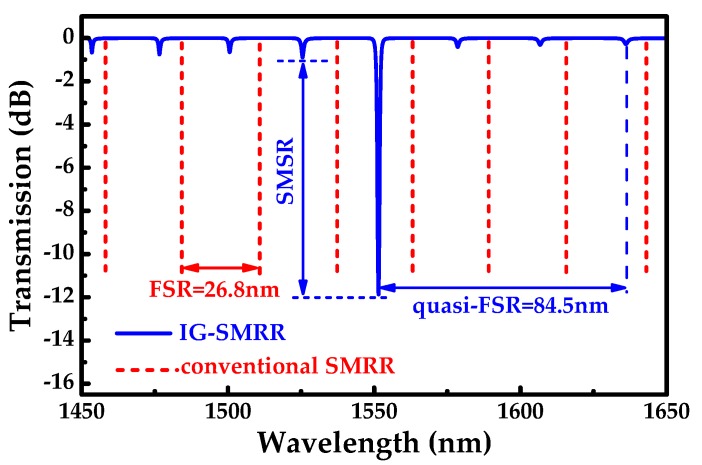
Transmission spectrum of the IG-SMRR (blue solid line) and conventional SMRR (red dashed line).

From the above optimization, several structural parameters of the sensor were determined: *W*_gap_ = 220 nm, *F* = 95%, and *H*_g_ = 18 nm. As shown in Figure 7, there is the only one main resonant peak at 1551.5 nm, from 1450 nm to 1650 nm. The first side mode to the left of the main resonant peak was evaluated by SMSR. The distance between the main resonant peak and its right third suppressed peak is denoted as the quasi-FSR. In the spectrum, the quasi-FSR of the IG-SMRR is 84.5 nm, and the FSR of conventional SMRR is 26.8 nm. For optical sensors, the achieved quasi-FSR is sufficient, which is the detection range of the IG-SMRR. Enlarged factor can be defined as the expression (9) to measure the broadening degree of detection range. Apparently, the enlarged factor is up to 3.15. Hence, the operating range of the proposed IG-SMRR gets effectively expanded. However, it is worth mentioning that the IG-SMRR has larger quasi-FSR (only one main resonator from 1400 nm to 1700 nm), which can be further investigated with the potential in other applications.

(11) $$A_{\mathrm{EF}}=\mathrm{quasi}-\mathrm{FSR}/\mathrm{FSR}$$

**Figure 8:**
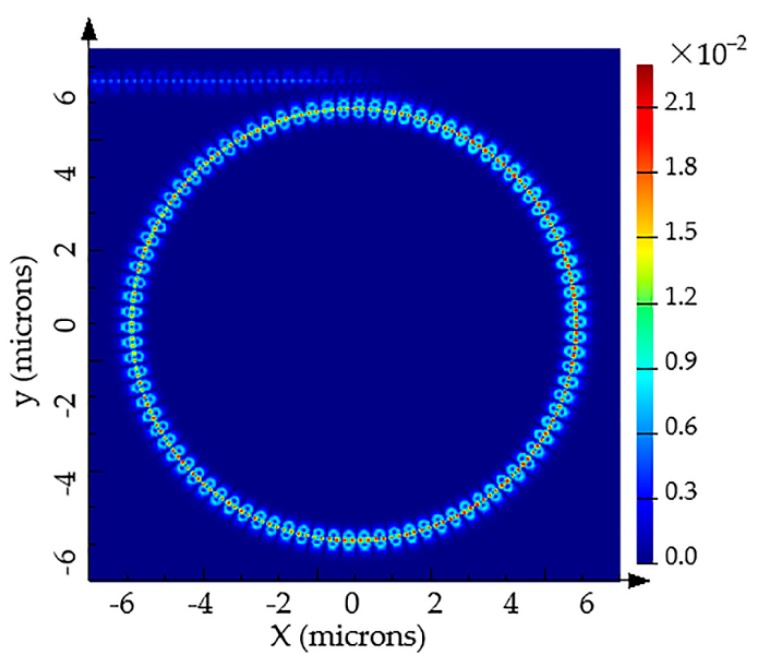
Power density distribution of the IG-SMRR at the resonant peak.

For the conventional SMRR sensors with the same width of ring waveguides, the center of mode field in the slot is partial to the outer ring due to the bend effect of ring waveguide, which leads to a certain amount of bending losses, so Q-factor gets decreased. Meanwhile the sensitivity becomes lower with the weakness of light–matter interaction. Therefore, designing asymmetric ring structure (inner ring width > outer ring width) enables resonant light to travel along the center of a slot. The introduction of IG to SMRR increases the inner ring width, which can play the same role. Power density distribution of the IG-SMRR at the resonant peak is shown in Figure 8, as can be seen from which that the resonant light lies in the center of the slot.

### 3.2. Deviation Analysis of the H_g_ and l_g_ in the Fabrication Process

**Figure 9:**
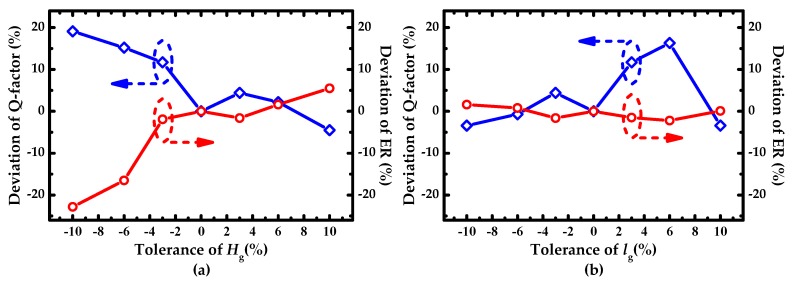
Effect of deviations of (**a**) *H*_g_ (corrugation depth) and **(b**) *l*_g_ (azimuthal width) on Q-factor and ER.

In the fabrication process of the device, the manufacturing tolerances can result in the fluctuation of the sensing performance for IG-SMRR. The deviations of Q-factor and ER induced by the tolerances of the corrugation depth *H_g_* and azimuthal width *l*_g_ are investigated. The tolerance range of *H*_g_ and *l*_g_ was set as −10%~10%, and deviations of Q-factor and ER are shown in Figure 9. As shown in Figure 9 a, Q-factor and ER fluctuate greatly in the variation range of −10%~−3% and manifest steady in the other range, which is acceptable. In Figure 9b, ER has minor fluctuation in the whole range, while Q-factor changes slightly in the variation range of −10%~0% and appears a peak in the positive direction for the rest of the range. The positive change of Q-factor is beneficial to IG-SMRR. However, oversize azimuthal width *l*_g_ can bring the difficulty in the fabrication of the corrugation width of IG. In a conclusion, the tolerance of *H*_g_ should be controlled as −3%~10%, and the tolerance of *l*_g_ is acceptable for the whole range −10%~10%.

### 3.3. Bulk Sensing Analysis

The sensing principle of IG-SMRR is similar to the optical waveguides. The aqueous solution concentrations as the top cladding are proportional to their RIs. The RI variations of aqueous solution influence the mode optical field interacting the surrounding samples in the slot of the IG-SMRR, which can result in the resonant effective RI variations. Here, the slot plays a crucial role in the sensing process due to the much resonant optical field in it. According to the resonant equation of MRR, the effective RI variations can induce the resonant wavelength shifts. Therefore, the relationship between the solution concentration change and the resonant wavelength shift can be built. The concentration sensitivity [26] of the optical sensor can be defined as:

(12) $$S_{C}=\Delta \lambda_{res}/\Delta C$$

where ΔC and $\Delta \lambda_{res}$ are the variations of sample (gas or liquid) concentration and resonant wavelength, respectively. This sensitivity means the resonant wavelength shift induced by 1% mass concentration change.

For comparative purposes, the universal RI sensitivity can be marked as:

(13) $$S_{V}=\Delta \lambda_{res}/\Delta n$$

where Δ*n* is the RI variation of aqueous solution. This sensitivity with the unit of nm/RIU means the resonant wavelength shift induced by unit RI change.

To demonstrate the bulk sensing characteristic of the IG-SMRR, sodium chloride (NaCl) and D-glucose (C_6_H_12_O_6_) were designated as samples. Their RIs with different aqueous solution concentrations (mass %) are listed in Table 1 [27].

**Figure 10:**
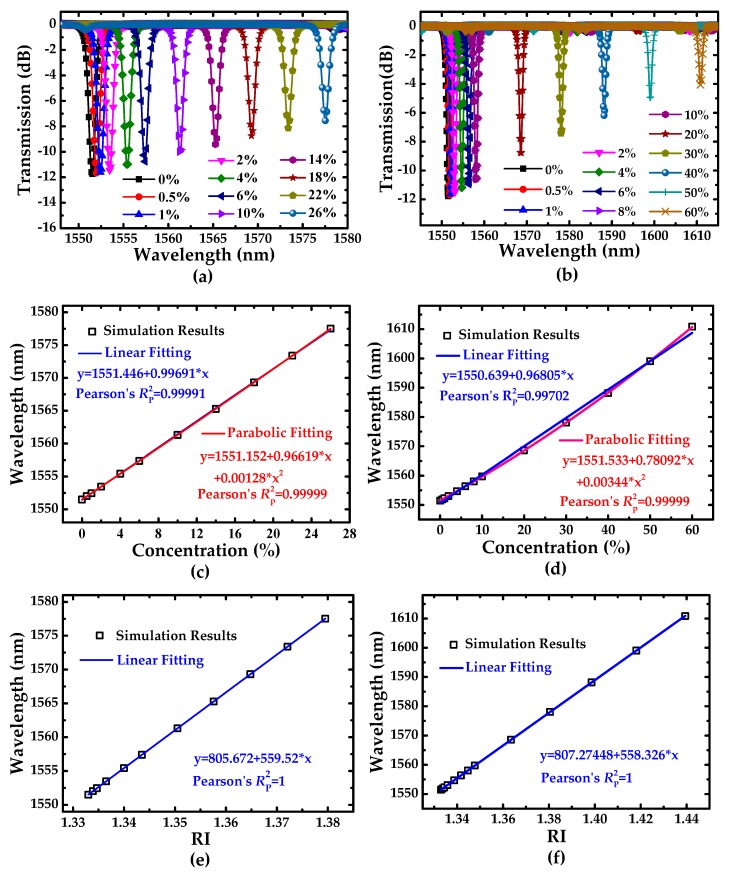
Transmission spectra of the IG-SMRR, the fitting curves of concentration sensitivity, and the fitting curves of RI sensitivity, the corresponding (**a**), (**c**), and (**e**) for sodium chloride solution, and the corresponding (**b**), (**d**), and (**f**) for D-glucose solution, respectively.

The conventional MRR can only detect the lower concentration range (<20%) [28]. The IG-SMRR can detect the higher concentration (>20%) of the solution due to the large quasi-FSR. The transmission spectra for different concentrations of sodium chloride solutions and D-glucose solutions are shown in Figure 10a,b, respectively. Figure 10b,d illustrates the function relationship between the resonant wavelength and the solution concentration. We utilize the linear and parabolic fittings of the simulation results to discuss the sensing sensitivity and linearity. As shown in Figure 10b,d, the blue solid and red dashed lines represent the linear and parabolic fittings for the simulation results. A redshift of the resonant wavelength can be observed with the increase of solution concentration. In addition, with the increase of the RIs of samples, the lower index contrast between the solution and the waveguide interfaces results in the smaller intrinsic loss of the waveguide, which induces the decrease of ER [2].

The slope of linear fitting represents the concentration sensitivity of the sensor. The coefficient of determination ($R_{P}^{2}$) is utilized to evaluate the quality of the fitted lines. The sensitivity and $R_{P}^{2}$ are listed in Table 2.

As concluded in Table 2, the concentration sensitivities of the sensor for sodium chloride solutions and D-glucose solutions are 996.91 pm/% and 968.05 pm/%, respectively. Compared with [19], both solution concentrations are over 10 times that of 95.27 pm/% and 95.33 pm/%, respectively. The $R_{P}^{2}$ for linear fit is less than the $R_{P}^{2}$ for parabolic fit, which illustrates that the 2 order parabolic fitting can depict the function relationship between the resonance wavelength and solution concentration more precisely than linear fitting [19,28,29,30,31]. For the low concentration variations of sodium chloride solutions, $R_{P}^{2}$ for linear fit is 0.99991, which shows the relationship between the resonance wavelength and solution concentration is nearly linear. However, for high concentration range of D-glucose solution, parabolic fitting can be considered. For the concentration sensitivity, a conclusion can be drawn that the linear fitting and parabolic fitting are suitable for the smaller range and larger range concentration detections, respectively.

In the field of the optical label-free sensor, RI sensitivity is employed more commonly for evaluating the sensing performance. Figure 10e,f illustrates the function relationship between the resonant wavelength and the RI for different solution concentrations. The RI sensitivities of the sensor for sodium chloride solutions and D-glucose solutions are 559.5 nm/RIU and 558.3 nm/RIU, respectively. Comparatively, the RI sensitivity demonstrates better linear characteristic in terms of the coefficient of determination $R_{P}^{2}$ = 1 for both aqueous solutions. 

## 4. Conclusions

In this work, the SOI IG-SMRR with an ultra-large detection range and a high sensitivity is proposed for label-free sensing. It combines SMRR with IG to enlarge the operating range due to good suppression of side modes. The related parameters are simulated and optimized to get the favorable transmission spectrum. The sensing device based on IG-SMRR has an ultra-large detection range. The concentration sensitivities of sodium chloride solutions and D-glucose solutions are 996.91 pm/% and 968.05 pm/%, respectively. And the RI sensitivities for the corresponding liquids are 559.5 nm/RIU and 558.3 nm/RIU, respectively. For the concentration sensitivity, the numerical analysis shows the linear and parabolic fitting are suitable for low concentration and high concentration detections, respectively. And RI sensitivity has better linear characteristic than concentration sensitivity. The proposed sensing device with a compact footprint of less than 13 μm × 13 μm is easily integrated with other SOI devices and enables integrated sensor arrays. Therefore, the SOI IG-SMRR combining the benefits of both SMRR and IG is a valuable exploration for micro/nano optical sensing applications in future.

## Figures and Tables

**Table 1:** RIs (refractive index) of the samples (at a temperature of 20 °C).

Sample	Concentration (%)	RI
Pure water		1.333
Sodium chloride	0.5–26	1.3339–1.3795
D-glucose	0.5–60	1.3337–1.4394

**Table 2:** Performance of the sensor with different samples for sensing application. $R_{P}^{2}$: coefficient of determination. ***S*_c_**: concentration sensitivity.

Sample	*S*_c_ (pm/%)	$R_{P}^{2}$ for Linear Fit	$R_{P}^{2}$ for Parabolic Fit
Sodium Chloride	996.91	0.99991	0.99999
D-glucose	968.05	0.99702	0.99999
